# Incorporating dose–volume histogram parameters of swallowing organs at risk in a videofluoroscopy-based predictive model of radiation-induced dysphagia after head and neck cancer intensity-modulated radiation therapy

**DOI:** 10.1007/s00066-020-01697-7

**Published:** 2020-10-09

**Authors:** Stefano Ursino, Alessia Giuliano, Fabio Di Martino, Paola Cocuzza, Alessandro Molinari, Antonio Stefanelli, Patrizia Giusti, Giacomo Aringhieri, Riccardo Morganti, Emanuele Neri, Claudio Traino, Fabiola Paiar

**Affiliations:** 1 Department of Radiation Oncology, University Hospital S. Chiara, Via Roma 55, 56100 Pisa, Italy; 2 Department of Physics, S. Luca Hospital, Via Guglielmo Lippi Francesconi 556, 55100 Lucca, Italy; 3 Department of Physics, University Hospital S. Chiara, Via Roma 55, 56100 Pisa, Italy; 4 Department of Radiation Oncology, University Hospital Cona, Via Aldo Moro 8, 44124 Ferrara, Italy; 5 Department of Radiology, University Hospital S. Chiara/Cisanello, Via Roma 55/Via Paradisa 2, 56100 Pisa, Italy; 6 Department of Clinical and Experimental Medicine, Section of Statistics, Via Roma 55, 56100 Pisa, Italy; 7 Department of Radiation Oncology, Ecomedica Institute of Clinical Research, Via Cherubini 2/4, 50053 Empoli, Italy

**Keywords:** Machine learning machines, Normal tissue complication probability, Aspiration, Deglutition, Radiotherapy

## Abstract

**Purpose:**

To develop a videofluoroscopy-based predictive model of radiation-induced dysphagia (RID) by incorporating DVH parameters of swallowing organs at risk (SWOARs) in a machine learning analysis.

**Methods:**

Videofluoroscopy (VF) was performed to assess the penetration-aspiration score (P/A) at baseline and at 6 and 12 months after RT. An RID predictive model was developed using dose to nine SWOARs and P/A-VF data at 6 and 12 months after treatment. A total of 72 dosimetric features for each patient were extracted from DVH and analyzed with linear support vector machine classification (SVC), logistic regression classification (LRC), and random forest classification (RFC).

**Results:**

38 patients were evaluable. The relevance of SWOARs DVH features emerged both at 6 months (AUC 0.82 with SVC; 0.80 with LRC; and 0.83 with RFC) and at 12 months (AUC 0.85 with SVC; 0.82 with LRC; and 0.94 with RFC). The SWOARs and the corresponding features with the highest relevance at 6 months resulted as the base of tongue (V65 and D_mean_), the superior (D_mean_) and medium constrictor muscle (V45, V55; V65; D_mp_; D_mean_; D_max_ and D_min_), and the parotid glands (D_mean_ and D_mp_). On the contrary, the features with the highest relevance at 12 months were the medium (V55; D_min_ and D_mean_) and inferior constrictor muscles (V55, V65 D_min_ and D_max_), the glottis (V55 and D_max_), the cricopharyngeal muscle (D_max_), and the cervical esophagus (D_max_).

**Conclusion:**

We trained and cross-validated an RID predictive model with high discriminative ability at both 6 and 12 months after RT. We expect to improve the predictive power of this model by enlarging the number of training datasets.

## Introduction

Radiation-induced dysphagia (RID) represents the real Achille’s heel of radiation-based organ preservation treatment in head and neck cancer (HNC). It contributes to a malnutritional status often requiring enteral nutrition and an increased risk of life-threatening aspiration pneumonia [1].

During and shortly after radio- (RT) or radiochemotherapy (RTCT), almost all HNC patients suffer from a certain degree of acute dysphagia mainly due to the high grade of mucositis and laryngeal edema that usually recovers within 3–4 months after treatment. However, in some cases, dysphagia may deteriorate over time (beyond 6 months) due to progressive fibrosis of the upper aerodigestive tract that, together with muscular disuse (usually due to the absence or significant reduction of oral intake), leads to permanent fibroatrophic damage causing many patients to suffer from a certain degree of RID for years after treatment [2, 3].

To date, clinicians are unable to accurately predict which patients will experience long-term RID. Recently, a multivariable clinical normal tissue complication probability model (NTCP) of swallowing dysfunction at 6 months following RT was externally validated by correlating the grade of dysphagia according to the Radiation Therapy Oncology Group (RTOG) and European Organization for Research and Treatment of Cancer (EORTC) late radiation morbidity score (a clinical primary endpoint) and average mean dose (D_mean_) to the Swallowing Organs at Risk (SWOARs) and salivary glands, reporting a good overall performance, discrimination, and goodness of fit [4]. In this regard, five patterns of clinical swallowing dysfunction related to RT doses to the upper and lower pharyngeal region as well as to the larynx and salivary glands were revealed (low, intermediate, and severe persistent; transient and progressive), suggesting different underlying radiobiological mechanisms [5].

SWOARs-sparing Intensity and Modulated Radiotherapy (SWOARs-IMRT) might reduce the probability of RID by generating highly conformal dose distributions that avoid these critical structures, thus achieving better functional outcomes [6]. Despite the small amount of data available in the current literature, a significant reduction of grade 2–4 swallowing dysfunction at 6 months after treatment has been observed by using SWOARs-sparing compared with a standard parotid-sparing IMRT [4]. We recently reported our prospective longitudinal study on nasopharyngeal and oropharyngeal cancers to assess the impact of RT or RTCT on swallowing function through an objective instrumental assessment from before treatment to 6 and 12 months after treatment using SWOARs-sparing IMRT (primary endpoint). We proved that better swallowing outcomes can be achieved with respect to standard approaches [7]. However, at present, it is unknown which SWOARs mostly contribute to RID so the prediction of its occurrence as well as its correlation with specific RT dose features is lacking.

Therefore, based upon the secondary endpoint of our study aimed to correlate objective instrumental swallowing outcomes with radiation dose absorbed by the SWOARs, we developed predictive model of RID from a set of RT dose metrics by using machine learning methodology. These classification algorithms have recently been promoted in radiology and are also increasingly being used in the radiotherapy field [8, 9]. In the study by Dean et al. [10], these methods were applied with good results to predict the occurrence of severe acute mucositis resulting from HNC RT with the aim of determining a predictive model for this side effect. However, at present, a predictive model of RID has not yet been created using machine learning methodology.

In this regard, the purpose of this research was to obtain a VF-based predictive model for RID based upon our abovementioned study by introducing SWOARs into the dose–volume histogram (DVH) analysis and by using a machine learning approach. In particular, the aims of this work were: 1) to design a classification framework to predict, with good performance, the onset of dysphagia from the DVHs of SWOARs; 2) to develop a predictive model of RID using a machine learning approach; 3) to identify the most important SWOARs and dosimetric features implicated in RID in order to guide treatment planning optimization.

## Materials and methods

### Patients, radiotherapy planning, and dysphagia assessment

Details of patients, radiation treatment characteristics, and the technical aspects of the instrumental swallowing evaluation have been reported previously [11].

Briefly, eligibility criteria included all patients affected by naso- and oropharyngeal cancers (stage II-IVA) who were candidates for RT or RTCT with curative intent requiring bilateral neck irradiation. Patients who had undergone prior induction CT or HN treatment (surgery and/or RT) or those with a diagnosis of concomitant comorbidity that might have compromised basic deglutition function (demyelinating or degenerative diseases and connective tissue diseases) were excluded to specifically address the impact of RT on swallowing function. Radiotherapy planning was optimized to reduce dose to the SWOARs (SWOARs-sparing IMRT). These were defined according to Christianen et al.’s guidelines [12]: the superior, middle, and inferior constrictor muscles (SPCM, MPCM, and IPCM); the supraglottic (SL) and glottic larynx (GL); cricopharyngeal muscle (CPM); cervical esophagus (CE); and parotid glands (PGs). In this regard, target coverage replaced sparing of any SWOARs except for the spinal cord in the IMRT optimization cost function. The IMRT plans set target prescription goals and spinal cord maximum dose as the highest priority, whereas SWOAR constraints were set as secondary.

According to the study protocol, an objective instrumental dysphagia assessment including VF was performed to assess the penetration-aspiration score (PAS score) at baseline and at 6 and 12 months after treatment. Two different consistencies were used to test the PAS score: 10 mL of thin barium (L = liquid) and 10 mL of paste barium (S = solid). To simplify the interpretation of data, PAS scores were divided into three different categories (1 = normal; 2–5: penetration; 6–8: aspiration) and dichotomized (0 = normal vs. 1 = penetration or aspiration).

Models of RID were developed using RT dose to the nine SWOARs and the occurrence of penetration or aspiration (P/A) at 6 and 12 months after treatment.

### Feature extraction

RT dose distributions for each patient were extracted using the TPS Eclipse^TM^ version 8.6 (Varian Medical Systems; Palo Alto USA). The RT dose distribution was described by differential and cumulative dose–volume histograms (DVHs) of SWOARs. Feature extraction was performed with a pipeline specifically designed using CERR [3] and MatLab software (R2017b; MathWorks, Inc.). The following eight features were extracted for each SWOAR from DVHs: V35, V45, V55, V65, minimum dose (D_min_), maximum dose (D_max_), mean dose (D_mean_) and the dose corresponding to the absolute maximum of the differential DVH, hence the modal dose (D_mp_). Therefore, a total of 72 features for each patient were calculated.

### Feature classification

Dose distribution features were analyzed using a machine learning procedure developed using MatLab software and Statistics and Machine Learning Toolbox (R2017b; MathWorks, Inc.). Three types of classification models were employed: linear support vector machines (SVMs) classifier, logistic regression, and classification ensembles. SVM classification (SVC) consists of a supervised binary classification method that learns the differences between two sample classes from a training set and uses a test set to quantify the classification performance on previously unseen data. In this analysis, as the number of features is quite high with respect to the number of examples, we only considered linear-kernel SVMs to prevent overfitting. SVMs are capable of handling very large feature spaces and have good generalization properties compared to conventional classifiers because in training the SVM classifier, the structural misclassification risk is to be minimized [13]. Training an SVM involves the estimation of the maximum margin hyperplane separating the training examples of the two groups. The training examples that fit in the margin are called support vectors. The separating hyperplane w • x + b = 0 is characterized by the weight vector w and the offset b (where w is a linear combination of the support vectors), and it is normal to the hyperplane [9].

In addition, logistic regression classification (LRC) was used to analyze DVH features. LRC is a linear method for predicting binary classes. The outcome in logistic regression analysis is dichotomous (0 or 1, negative or positive, respectively). In the multiple logistic regression model, the expected probability, P, that the outcome of interest is 1 can be written as follows:

1 $$\mathrm{P}=e^{f}/(1+e^{f})$$

where f = β_0_ + sum_i_(β_i_x_i_), x_1_ … x_p_ are the p training examples, β_0_ is the bias, and β_1_ … β_p_ the estimated coefficients [14]. This model is particularly useful in classical radiobiology to describe NTCP.

Finally, the classification ensemble method was used to study the discriminative power of the DVH features of SWOARs. Ensemble-learning algorithms are supervised classification methods that aggregate weaker learners into one high-quality ensemble predictor. The bootstrap aggregation is a technique for reducing the variance of an estimated prediction function. It is used to bag a weak learner such as a decision tree on the dataset to generate many bootstrap replicas and grow decision trees on them. Each bootstrap replica is obtained by a random selection of m training examples out of *N* with replacement, where *N* is the dataset size. Moreover, every tree in the ensemble can randomly select predictors for each decision split. This technique is called random forest classification (RFC) and it improves the accuracy of bagged trees. Usually, the number of examples to select for each split is equal to the square root of the number of predictors *p* used for classification [14–16].

Performances are evaluated in terms of sensitivity (true positive ratio, i.e., the percentage of positive subjects correctly classified) and specificity (i.e., the proportion of negatives that are correctly identified as such). The trade-off between the sensitivity and the false-positive ratio (one minus the test specificity), obtained by varying the decisional threshold of the classifier, is known as the receiver operating characteristic (ROC) curve [17]. From the ROC curve, the area under curve (AUC) can be estimated. The AUC is a global index to compare the ROC curves of different classifiers [18]. In all the classification analyses, AUC was computed in cross-validation (CV), both to guarantee an unbiased estimate of the classifier performance and to achieve a good generalizability of the models. The classifiers were trained according to the leave-one-out cross-validation (LOO-CV) technique, which is usually implemented when a rather limited dataset is available. This method performs *N* repetitions, where *N* is equal to the number of subjects in the dataset. At each iteration, the training set is composed of all the examples minus one (*N* - 1 subjects), which is used to test the trained model. As a consequence, the test data never enter the training phase. The prediction results from the *N* classifiers can be combined in order to get an unbiased estimate of the AUC of the classifier, despite the poor number of examples [19–21]. In order to estimate AUC range, a nested LOO-CV was applied. In each of *N* iterations a further LOO-CV was implemented on the *N* - 1 training examples to compute AUC.

### Feature reduction

In this study, a data dimensionality reduction procedure was applied with the aim of identifying the features with the highest discriminative power. Features were ranked by relevance using out-of-bag predictor importance estimates by permutation. This method measures how influential the predictor variables in the model are at predicting the response. The influence of a predictor increases with the value of this measure. If a characteristic is influential in prediction, then permuting its values should affect the model error. If a predictor is not influential, then permuting its values should have little to no effect on the model error. Out-of-bag (OOB) error is the mean prediction error on each training sample *x*_*i*_, using only the trees that did not have *x*_*i*_ in their bootstrap sample. The omitted observations are called OOB observations [16]. A permutation algorithm can be used, permuting OOB data across one predictor at a time and estimating the increase in OOB error due to this random permutation. With this method a reliable estimate of feature importance can be obtained in the training phase.

## Results

Between June 2012 and October 2015, 39 patients with nasopharyngeal (*n* = 10) and oropharyngeal (*n* = 29) cancer were enrolled in our study. 38 were eligible for the evaluation of the study, as 1 patient died due to cardiovascular disease at 4 months after treatment. Of the 38 eligible patients, 36 and 30 patients underwent VF at 6 and 12 months, respectively. The summaries of baseline patient and tumor characteristics and treatment details are reported elsewhere [7]. Among the 38 evaluable patients, 7 (18%) experienced a locoregional recurrence (6 local and 1 both local and regional), of whom 5 underwent subsequent metastatic progression (4 lung and 1 bone metastasis). Contrastingly, 2 patients (1 nasopharynx and 1 base of tongue) experienced distant metastases progression without locoregional evidence of recurrence.

P was found in 6/36 patients (17%) and A in 5/36 patients (14%) at 6 months after treatment, and in 6/30 patients (20%) and 3/30 patients (10%), respectively, at 12 months after treatment. Among these patients, only 1 (3%) experienced clinical aspiration pneumonia which required hospitalization, protective tracheostomy, and antibiotic therapy.

Among the 11/36 patients (30.5%) who developed RID at 6 months, 6 patients (55%) still persisted at 12 months, 4 patients (36%) did not receive the exam at 12 months, whereas only 1 (9%) developed RID at 12 months and not at 6 months after treatment.

### Feature analysis with machine learning methods

In this study, dose distribution features of SWOARs were used as predictor variables in three supervised learning methods: linear-kernel SVC, LRC, and RFC. Patients were grouped on the basis of P/A patterns at 6 and 12 months: class label was set to 0 in case of normal deglutition and to 1 if the event of P or A occurred. Hence, two binary classifications were designed: at 6 months 0 vs 1 0 (number of patients [*N*] = 25) vs. 1 (*N* = 10) and at 12 months 0 vs. 1 0 (*N* = 20) vs. 1 (*N* = 9). In order to avoid scale-related dominance, each column of the predictor data was centered and scaled by the column mean and standard deviation, respectively.

The mean doses to SWOARs for patients grouped on the basis of P/A at 6 and at 12 months are summarized in Table 1.

**Table 1:** Mean doses of SWOARs for all patients based on the occurrence of RID (group 0 = normal swallowing vs. group 1 = penetration or aspiration) at 6 and 12 months after treatment

SWOARs	P/A 6 months		P/A 12 months	
	Group 0	Group 1	Group 0	Group 1
	Mean ± SD (range), Gy	Mean ± SD (range), Gy	Mean ± SD (range), Gy	Mean ± SD (range), Gy
*SPCM*	53 ± 15 (13–66)	61 ± 4 (53–67)	52 ± 16 (13–66)	57 ± 12 (29–67)
*MPCM*	49 ± 10 (30–62)	61 ± 4 (54–67)	48 ± 10 (30–61)	62 ± 5 (50–67)
*IPCM*	40 ± 13 (20–61)	53 ± 9 (39–67)	37 ± 13 (20–61)	56 ± 8 (42–67)
*SL*	45 ± 14 (20–68)	56 ± 10 (40–68)	44 ± 15 (20–68)	59 ± 11 (38–68)
*GL*	37 ± 14 (16–63)	47 ± 9 (37–67)	35 ± 14 (18–63)	51 ± 9 (38–67)
*CPM*	37 ± 12 (17–58)	48 ± 8 (38–61)	35 ± 12 (17–55)	51 ± 8 (40–61)
*CE*	24 ± 11 (2–42)	34 ± 10 (23–50)	23 ± 11 (2–42)	36 ± 9 (26–50)
*BOT*	52 ± 10 (30–64)	63 ± 2 (60–67)	50 ± 10 (30–64)	62 ± 4 (55–67)
*PGs*	25 ± 8 (9–41)	30 ± 5 (22–37)	24 ± 8 (9–42)	30 ± 6 (22–39)

*SWOARs* swallowing organs at risk, *RID* radiation-induced dysphagia, *P* penetration, *A* aspiration, *SD* standard deviation, *SPCM* superior constrictor muscle, *MPCM* medium constrictor muscle, *IPCM* inferior constrictor muscle, *SL* supraglottic larynx, *GL* glottic larynx, *CPM* cricopharyngeal muscle, *EC* cervical esophagus, *BOT* base of tongue, *PGs* parotid glands

The classifiers were trained according to the LOO-CV procedure, thus excluding one subject from the training set at each iteration and validating the trained model on it. Using this method it was possible to estimate AUC in cross-validation as a unique classification performance index. The range of AUC values for each classifier and for two subgroups at 6 and at 12 months was estimated with nested LOO-CV.

**Fig. 1:**
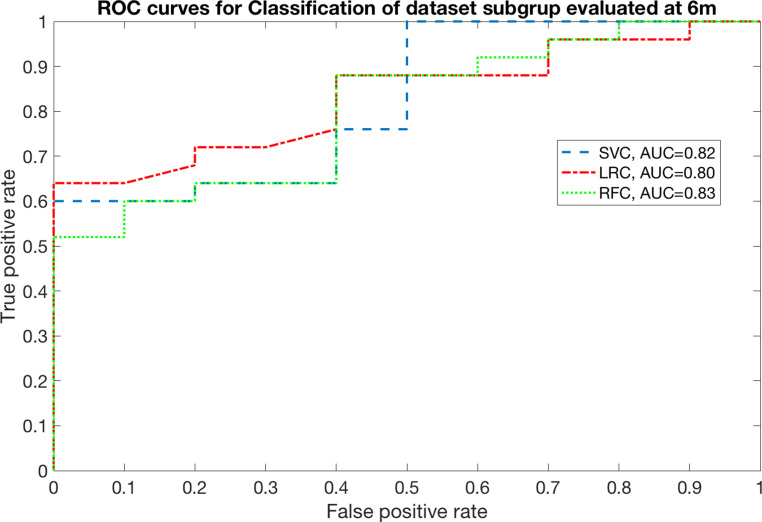
Receiver operating characteristics (ROC) curve for prediction of disturbed swallowing (penetration/aspiration) at 6 months

**Fig. 2:**
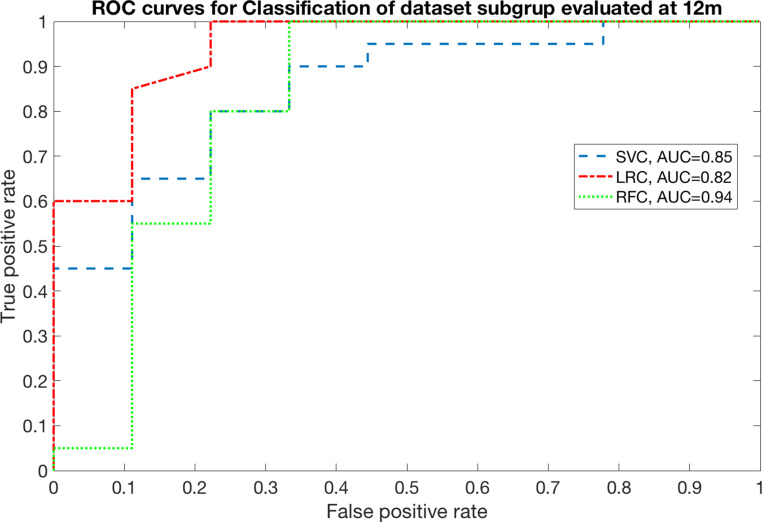
Receiver operating charactheristics (ROC) curve for prediction of disturbed swallowing (penetration/aspiration) at 12 months

The relevance of SWOAR DVH features in predicting RID emerged both at 6 and 12 months. At 6 months AUC was 0.82 with SVC (range [0.80–0.92]), 0.80 with LRC (range [0.74–0.88]), and 0.83 with RFC (range [0.79–0.93]). Moreover, at 12 months AUC was equal to 0.85 with SVC (range [0.82–0.95]), 0.82 with LRC (range [0.76–0.95]), and 0.94 with RFC (range [0.91–0.99]). The ROC curves for all the classifiers are reported in Figs. 1 and 2.

The application of LRC allows the estimation of logistic regression bias and parameters useful to obtain an NTCP model of radiation-induced dysphagia. Here, the model is multivariate and depends simultaneously on many dose–volume variables computed for a set of SWOARs (a total of 72 features). The model is given by the following: NTCP = e^f^ / (1 + e^f^), where f = β_0_ + sum_i_(β_i_x_i_), where β_0_ is the bias, and β_i_ is the estimated coefficient for the variable x_i_, centered and scaled. The quality of the model was evaluated by computing AUC with LOO-CV by combining the prediction results from the *N* logistic regression classifiers.

### Feature importance analysis

**Fig. 3:**
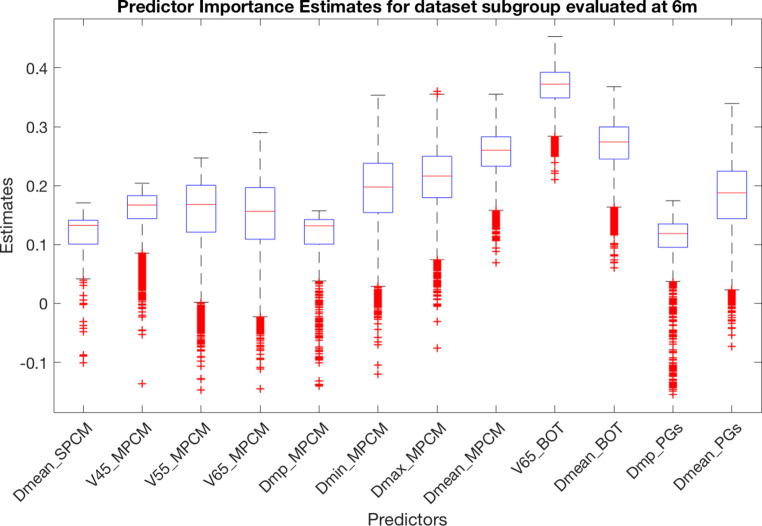
Predicator importance estimates for prediction of disturbed swallowing (penetration/aspiration) at 6 months

**Fig. 4:**
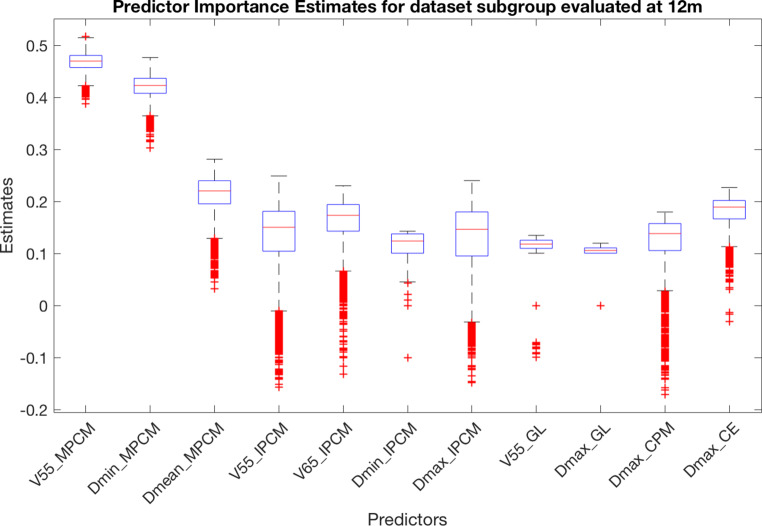
Predicator importance estimates for prediction of disturbed swallowing (penetration/aspiration) at 12 months

To understand which of the 72 characteristics are the most relevant in P/A subgroup discrimination, an OOB permutation algorithm was implemented both at 6 and at 12 months. A number of 10,000 iterations was set and the results are shown in Figs. 3 and 4, where the features with an estimated importance >0.1 are reported. The SWOARs and the corresponding features with the highest relevance level in predicting RID at 6 months were the BOT (V65 and D_mean_), SPCM (D_mean_), MPCM (V45, V55, V65, D_mp_, D_mean_, D_max_, and D_min_), and PGs (D_mean_ and D_mp_). On the contrary, of highest importance at 12 months were MPCM (V55, D_min_ and D_mean_), IPCM (V55, V65, D_min_, and D_max_) together with GL (V55 and D_max_), CPM (D_max_) and EC (D_max_).

## Discussion

The purpose of this study was to provide a predictive model of RID based on VF findings at 6 and 12 months on naso- and oropharyngeal patients after definitive RT or RTCT using data from a prospective study conducted in our institution between June 2012 and July 2015.

Our previously published results [7] showed a significant worsening of VF-assessed swallowing function for S (*p* = 0.039) rather than L bolus (no statistically significant variations) reporting an overall pattern of post-swallowing P/A of 31% and 30% at 6 and 12 months after treatment. Specifically, P was found in 6 patients (17%) and A (14%) in 5 patients (14%) at 6 months, and in 6 patients (20%) and 3 patients (10%) at 12 months after treatment, respectively. With these data, we built a predictive model of RID by introducing nine SWOARs into the DVH analysis and extracting eight different dosimetric features for each SWOAR in order to implement a machine learning analysis.

At first, we found the model to be highly accurate in predicting the occurrence of RID both at 6 and 12 months after treatment, reporting an AUC of 0.82 with SVC and of 0.83 with RFC at 6 months and of 0.85 with SVC and of 0.94 with RFC at 12 months. Specifically, we discovered that the dose received by the SWOARs located in the upper HN region (SPCM, BOT, MPCM, and PGs) was extremely important for predicting RID at 6 months, whereas the dose received by the SWOARs located in the lower region (IPCM, GL, CPM, and EC) was extremely important for predicting RID at 12 months after treatment. These findings seem to reflect different pathophysiological mechanisms in the impairment of pharyngeal swallowing phase due to the involvement of different swallowing structures early and late after treatment. As widely reported, there is a strict correlation between the severity of post-swallowing residue and the risk of P/A after an RT-based treatment [22, 23], and the restriction of the pharyngeal craniocaudal driving pressure, together with the reduction of the back–forward movement of the BOT, might explain our early results. Thus, the persistence of high-grade inflammatory edema of both the upper pharyngeal musculature and the BOT, worsened by the RT-related xerostomia (due to parotid glands damage), may cause a high-grade post-swallowing pharyngeal residue that leads to spillage of the bolus into the airways. Nevertheless, due to the lack of statistical significance of these SWOARs at 12 months after treatment, our results seem to determine a reversible effect of RT on these structures, probably suggesting that objective swallowing dysfunction at 6 months is related to prolongation of acute effects rather than to the onset of late effects. Therefore, the recovery or at least improvement of the salivary function through the frequent use of substitute or stimulator products and early deglutitory rehabilitation (mostly aimed at strengthening the retropulsion movement of the BOT), together with the total or partial recovery of oral nutrition in this time interval, might explain the impairment retrieval for these swallowing structures between 6 and 12 months [24–29]. On the contrary, our findings seem to identify different mechanisms of late RID, because the SWOARs located in the lower part of the HN region seem to be those mostly implicated in the occurrence of this complication. In our experience, the radiation damage of the most caudal SWOARs played a crucial role in the late swallowing impairment of our patients. These findings are likely explained by a reduction of the protective mechanisms of the airways (mainly guaranteed by the early closure of the true and false vocal cords as well as by the hyolaryngeal complex elevation) due to post-RT laryngeal damage and by the hampered opening of the upper aerodigestive tract (guaranteed by the relaxation of the cricopharyngeal muscle and upper esophageal sphincter) due to CPM and EC post-RT damage. In addition to the above-described mechanisms, the radiation-induced fibroatrophic damage of the MPCM and IPCM probably contributes to impairing the craniocaudal pharyngeal peristalsis, leading to an increase of post-swallowing pharyngeal residue and subsequent inhalation [30–33].

The histological and biochemical structures of the pharyngeal tract seem to support our findings. A higher sensitivity to radiation damage for the lower rather than the upper pharyngeal muscular tract (due to a different myofiber composition for the inner and outer layers) has been reported by the literature [34–37]. As a matter of fact, the inner layer is composed of a high prevalence of myofibers containing slow-twitch myosin (type I) characterized by slow contraction, high mitochondrial density, and high oxidative capacity, and is mostly responsible for the tone and stiffness of the pharynx. On the contrary, the outer layer is composed of a high prevalence of myofibers containing fast-twitch myosin (Type IIb) characterized by fast contraction, low mitochondrial density, and low oxidative capacity, and is mostly responsible for the contraction of the pharynx and the propulsion of the bolus. The immunohistochemical analysis of the pharynx, reported by Liancai et al. [35], has shown that the ratio between the width of the two layers changes in a craniocaudal direction, from approximately 2:1 in the cranial portion to 1:2 in the caudal portion. This might explain the higher radiosensitivity of the caudal compared to the cranial SWOARs and we believe this might further support our results.

To the best of our knowledge, this is the first analysis that aims to develop a predictive model of RID and combines a VF-based swallowing assessment with a quantitative differential analysis of DVH for nine different SWOARs using a machine learning method. The multivariate technique employed in this study challenges the common practice in RT planning in which single constraints for each organ at risk are considered during treatment plan optimization. Here, the DVH characteristics of nine SWOARs are taken as a unique object, all contributing at the same time to the RID predictive model. In this regard, the study by Eisbruch et al. [38] was the only one that specifically investigated the relationship between VF-based aspiration and dose received by the pharyngeal constrictor muscles and the supraglottic larynx in a prospective experience on 73 oropharyngeal cancer patients. In this study, mean doses to each part of the PC (superior, middle, and inferior) were significantly correlated with all VF dysphagia measures, with superior PCs demonstrating the highest correlations. Interestingly, the lack of a mean dose threshold for these swallowing-related structures, as well as toxic doses (TD_25_) of 56 Gy and 39 Gy, were detected for pharyngeal constrictors and supraglottic larynx, respectively.

Despite the fact that our results are very encouraging in terms of predictive performances, we know that a limitation of our analysis is the low number of patients. Regarding this point, it is not unusual to find machine learning studies with rather small datasets [20, 21], especially if data are robust and well characterized. Nevertheless, in these cases reliable results can also be achieved providing that the method is chosen adequately. For this reason, in our study we decided to implement LOO-CV, both to guarantee an unbiased estimate of classification performances and to maximize the training set size at each iteration. In fact, LOO-CV offers a chance of squeezing the maximum out a small dataset and obtaining an estimate of AUC that is as accurate as possible [19]. Secondly, we know that the main limitation of our analysis is the absence of an external validation. However, we expect to improve the generalization capability of this model both by enlarging the number of examples and by adding a group of patients to use for external validation only. In this regard, we intend to validate the RID predictive model once the same data from the larger dataset of the ongoing Italian Multicentric Clinical Study (NCT 03448341) are available. In the meantime, we strongly suggest that clinicians and physicists maximally enforce the dosimetric constraints of the lower SWOARs in the IMRT plan optimization process for this specific subset of HNC patients in order to reduce the risk of late RID. Using a clinical primary endpoint, the influence of RT doses to inferior SWOARs (especially to the inferior and medium constrictor muscles) in oropharyngeal patients to determine late RID has been recently reported by Mogadas et al. [39].

Last but not least, we would like to point out the considerable potential of a machine learning methodology. This new approach builds upon NTCP models based on the combination of multiple dose metrics for different organs at risk. These are considered as a unique object rather than as a standard approach, in which dose values for each organ at risk are taken separately. If so, these NTCP models could be used in daily practice for treatment planning optimization and clinical decision support. We believe that this approach should be applied in radiation oncology research when the occurrence of an organ dysfunction, similarly to RID, is related to the damage of several rather, than just one, functional structures that differently concur in physiological processes.

## Conclusion

In this study we began to lay the foundations for a future investigation of the relevance of the different SWOARs in the occurrence of RID. Our results highlight a major critical role of the impairment of BOT and PGs for early RID, and of IPCM, CPM, GL, and CE for late RID. Also, a crucial role seems to be played by MPCM both for early and late RID, maybe due to its anatomical location between upper and lower HN region that makes it difficult to be spared for tumors located in upper HN region.

Therefore, we would like to draw the attention of the clinicians to the abovementioned SWOARs, several of which have been poorly considered until recently and are worth further investigating in clinical research.
